# Effect of pre-stroke statin use on the progression of intracranial atherosclerosis in ischemic stroke patients: Evidence from high-resolution magnetic resonance imaging

**DOI:** 10.1097/MD.0000000000045493

**Published:** 2025-12-26

**Authors:** Shibo Dong, Hongshan Chu, Yuan Zhang, Ruisheng Duan, Hongyu Hao, Xing Xing, Nan Yin, Jin An, Ya Gao, Xiangjian Xiao

**Affiliations:** a Department of Medical Imaging, Hebei General Hospital, Shijiazhuang, Hebei Province, China; b Department of Neurology, Hebei General Hospital, Shijiazhuang, Hebei Province, China.

**Keywords:** high-resolution magnetic resonance imaging (HR-MRI), ischemic stroke, statin

## Abstract

High-resolution magnetic resonance imaging (HR-MRI) has emerged as a valuable tool for evaluating intracranial atherosclerotic plaque characteristics in vivo. This study aimed to identify clinical and imaging predictors of plaque enhancement (PE) on HR-MRI in patients presenting with acute ischemic stroke (AIS) or transient ischemic attack (TIA). We retrospectively included consecutive patients diagnosed with AIS or TIA who underwent HR-MRI between January 2021 and August 2022. Participants were categorized according to the presence or absence of contrast enhancement in culprit plaques and further stratified by prior statin usage. Plaque enhancement was defined as increased signal intensity on contrast-enhanced T1-weighted images. Clinical profiles and imaging parameters were compared between groups. Among the 208 participants, 138 demonstrated enhancement of the culprit plaque, whereas 70 showed no such enhancement. Enhanced plaques were associated with significantly greater plaque burden and higher degrees of luminal stenosis (both *P* < .001). At the site of maximum stenosis, patients without enhancement exhibited significantly larger vessel area (VA; *P* = .01) and lumen area (LA; *P* < .001). A modest but statistically significant correlation was observed between stenosis severity and enhancement grade (Spearman ρ = 0.372, *P* < .001). Notably, prior use of statins was associated with a lower incidence of PE (*P* = .03). HR-MRI–detected PE is associated with more severe arterial narrowing and may serve as an indicator of plaque instability. The observed reduction in enhancement among statin users supports the potential role of statin therapy in stabilizing intracranial atherosclerotic lesions.

## 1. Introduction

Intracranial atherosclerosis (ICAS) is a major cause of ischemic stroke, particularly in Asian populations, where it accounts for approximately 10% of transient ischemic attacks (TIA) and 30 to 50% of ischemic strokes.^[1]^ The mechanisms by which ICAS contributes to stroke include thrombotic occlusion of perforating arteries, artery-to-artery embolism, and hemodynamic compromise due to severe stenosis.^[2–4]^ Conventional imaging modalities, such as magnetic resonance angiography (MRA) and computed tomography angiography, mainly provide information on luminal narrowing but cannot adequately characterize the underlying vessel wall pathology. In contrast, high-resolution magnetic resonance imaging (HR-MRI) enables detailed evaluation of plaque morphology and biological activity, offering additional insights into plaque vulnerability.^[5,6]^ Among HR-MRI markers, plaque enhancement (PE) after contrast administration has been recognized as a surrogate for inflammatory activity and instability.

Previous studies have suggested that metabolic risk factors, including dyslipidemia, are associated with PE.^[7]^ Given that statins exert both lipid-lowering and anti-inflammatory effects, their potential to stabilize intracranial atherosclerotic plaques has become an important area of investigation. However, evidence regarding the relationship between pre-stroke statin use and PE remains limited. Therefore, the present study specifically aimed to evaluate whether prior statin therapy is associated with reduced PE in patients with acute ischemic stroke (AIS) or TIA, thereby exploring the potential role of statins in stabilizing intracranial atherosclerotic lesions.

## 2. Materials and methods

### 2.1. Patient selection and study design

This study was approved by the Ethics Committee of Hebei General Hospital (Approval Number: 2021-KY-123). This cross-sectional retrospective study was carried out in the Department of Neurology at Hebei General Hospital from January 2021 to August 2022, enrolling 208 consecutive patients. Eligible participants had experienced an AIS or TIA within 7 days of onset, attributed to symptomatic ICAS, and had undergone HR-MRI during hospitalization. Exclusion criteria included: presence of cardioembolic sources such as emboligenic heart disease^[8]^; significant (≥50%) stenosis in relevant extracranial arteries due to extracranial atherosclerosis; nonatherosclerotic vascular conditions, including aortic dissection, moyamoya disease, vasospasm, vasculitis, or coagulopathies; and imaging data of a low quality.

### 2.2. Demographics and laboratory data

Demographic information and vascular risk factors were extracted from medical records. These included age, sex, body mass index (kg/m^2^), smoking status (defined as daily smoking of at least one cigarette for 6 consecutive months), diabetes mellitus (defined as fasting plasma glucose ≥ 7.0 mmol/L or use of antidiabetic medication), hypertension (systolic blood pressure ≥ 140 mm Hg, diastolic blood pressure ≥ 90 mm Hg, or current use of antihypertensive medication), and prior statin use (consistent statin therapy for more than one month before the index stroke). Laboratory data were also collected, including lipid profiles – total cholesterol, triglycerides, low-density lipoprotein cholesterol (LDL-C), high-density lipoprotein cholesterol（HDL-C）, non-HDL cholesterol, apolipoprotein B (ApoB), apolipoprotein A (ApoA), and the ApoB/ApoA ratio. Additional parameters included uric acid, blood urea nitrogen, creatinine, creatine kinase, homocysteine, serum total protein, albumin, fasting glucose, fibrinogen, D-dimer, white blood cell count, platelet count, hemoglobin, and glycated hemoglobin (hemoglobin A1c).

### 2.3. MR imaging

All imaging examinations were conducted using a 3.0 Tesla magnetic resonance imaging (MRI) scanner (Discover MR750w 3.0T; GE Medical Systems, LLC) by using a 24-channel head and neck joint coil for reception. Time-of-flight MRA was used to reconstruct vascular architecture and guide precise localization of the stenotic lesions of selected vessels. HR-MRI scanning was carried out, including black-blood coronal T1-weighted images, pre- and post-contrast. HR-MRI was conducted using the following imaging sequences and parameters. For time-of-flight-MRA, the repetition time (TR) was 19 ms, echo time (TE) was 2.9 ms, the number of excitations (NEX) was 1, the field of view (FOV) was 220 × 194 mm^2^, matrix size was 320 × 320, slice thickness was 1.0 mm, and a total of 84 slices were acquired. For T1WI, the TR was 1300 ms, TE was 16.7 ms, NEX was 1, FOV was 180 × 180 mm^2^, matrix size was 320 × 320, slice thickness was 0.6 mm, with no slice gap, and 124 slices were obtained. For diffusion weighted imaging, the TR was 7468 ms, TE was 77.2 ms, NEX was 2, FOV was 240 × 240 mm^2^, matrix size was 130 × 160, slice thickness was 4.0 mm, with no slice gap, and 72 slices were acquired. Post-contrast HR-MRI was performed approximately 5 minutes after intravenous injection of a gadolinium-based contrast agent (0.1 mmol/kg body weight), to allow sufficient time for vessel wall enhancement.

Two experienced neuroradiologists, blinded to the clinical data, independently assessed all HR-MRI measurements. Inter-observer agreement for PE grading was evaluated using Cohen κ coefficient. For continuous parameters (vessel area (VA), lumen area (LA), wall area (WA), and plaque burden), inter-observer agreement was analyzed with the intraclass correlation coefficient. The inter-observer agreement for PE grading was good (κ = 0.81, *P* < .001). The intraclass correlation coefficient values VA, LA, WA, and plaque burden ranged from 0.87 to 0.92, indicating excellent reproducibility.

### 2.4. Image analysis

All measurements were carried out using the GE Advantage Workstation. The neuroradiologist determined the specific vessel and site for assessment based on the patient’s symptoms. Atherosclerotic plaques were characterized on high-resolution MRI as areas of uneven wall thickening, with or without narrowing of the vessel’s lumen, and were observed in both precontrast and postcontrast reconstructed images. The plaque considered responsible for the stroke, referred to as the culprit plaque, was identified either as the only lesion within the affected vascular area or as the one causing the greatest narrowing when multiple plaques were present. This determination was made in accordance with the patient’s clinical symptoms as well as the neurologist’s evaluation. The coronal T1WI images were enlarged to 300% for measurement of VA and LA at the most narrowest lumen, where the thickest plaque was located, as well as at the reference site. The reference location was defined as the closest proximal vascular segment displaying minimal or no plaque burden. If a suitable proximal reference site was unavailable, an adjacent distal segment with minimal plaque was used instead. The WA was obtained by calculating the difference between the VA and the LA. To assess the extent of atherosclerotic involvement, plaque burden was quantified by dividing the WA by the total VA, providing a proportional measure of plaque accumulation within the vessel wall. The degree of stenosis was assessed using the formula: (1 – LA at the stenotic site/reference LA) × 100%. PE of the culprit lesion was also assessed. Enhancement was categorized as follows: grade 0, where the signal intensity was equal to or lower than the pituitary stalk’s signal intensity, grade 1, where the signal intensity was higher than grade 0 but lower than the pituitary stalk’s, and grade 2, where the signal intensity was equal to or higher than the pituitary stalk.^[9]^ Two experienced radiologists, blinded to the clinical data, independently conducted all measurements. The imaging slice chosen for analysis was selected through consensus between the 2 observers.

### 2.5. Statistical analysis

Statistical analysis was carried out using IBM SPSS software, version 26. Categorical data were presented as frequencies and percentages. All continuous variables were first tested for normality using the Shapiro–Wilk test. Normally distributed data were expressed as mean ± standard deviation, while non-normally distributed data were expressed as median (interquartile range). For comparisons involving continuous variables, the independent *t*-test or one-way ANOVA was applied for normally distributed data, whereas the Mann–Whitney *U* test or Kruskal–Wallis test was used for non-normally distributed data. Categorical variables were analyzed using either the χ^2^ test or Fisher exact test, as applicable. Spearman correlation coefficient was employed to assess the correlation between the degree of vascular stenosis and the extent of PE. Correlation strength was categorized as follows: *R* > 0.95 indicates a strong correlation, *R* ≥ 0.8 denotes a high correlation, 0.5 ≤ *R* < 0.8 suggests a moderate correlation, 0.3 ≤ *R* < 0.5 reflects a weak correlation, 0 < *R* < 0.3 implies no correlation, and *R* < 0 signifies a negative correlation. Multiple logistic regression analysis was conducted to determine independent predictors of PE and the remodeling index of ICAS as observed on HR-MRI, while controlling for potential confounding variables identified through univariable analysis (*P* < .05). For each covariate, odds ratios along with 95% confidence intervals were calculated. Statistical significance was defined as a two-tailed *P*-value < .05. Missing data were addressed by performing complete-case analysis, and patients with unavailable values were excluded from the relevant comparisons.

## 3. Resluts

### 3.1. Characteristics of patients with enhancing ICAS

**Figure 1.**
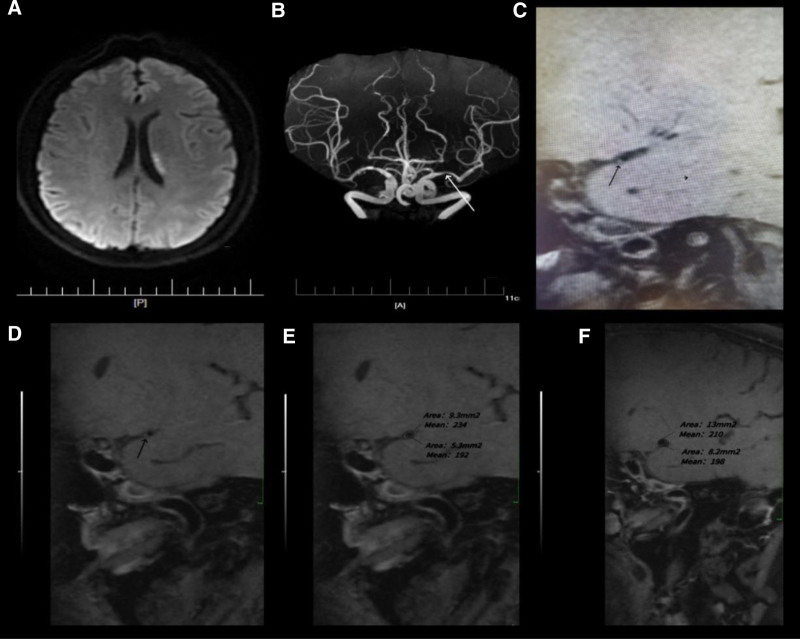
HR-MRI of the symptomatic middle cerebral artery (MCA) stenosis in a 67-yr-old female presenting with right upper limb weakness lasting 16 h. (A) Diffusion weighted imaging (DWI) reveals an acute ischemic infarct in the left MCA territory. (B) TOF-MRA demonstrates severe stenosis in the M1 segment of the left MCA (white arrow). (C) T1-weighted HR-MRI identifies the plaque (black arrow). (D) Post-contrast T1-weighted HR-MRI shows eccentric wall thickening without evident enhancement at the plaque site (black arrow). (E) At the point of maximal narrowing, post-contrast T1-weighted HR-MRI shows a V of 9.3 mm^2^ and an LA of 5.3 mm^2^. (F) The vessel area is 13.0 mm^2^ at the reference site, and the LA is 8.2 mm^2^. The degree of stenosis is calculated as: (1–5.3 mm^2^/ 8.2 mm^2^) × 100% = 35.37%. HR-MRI = high-resolution magnetic resonance imaging, LA = lumen area, TOF-MRA = time-of-flight magnetic resonance angiography.

**Figure 2.**
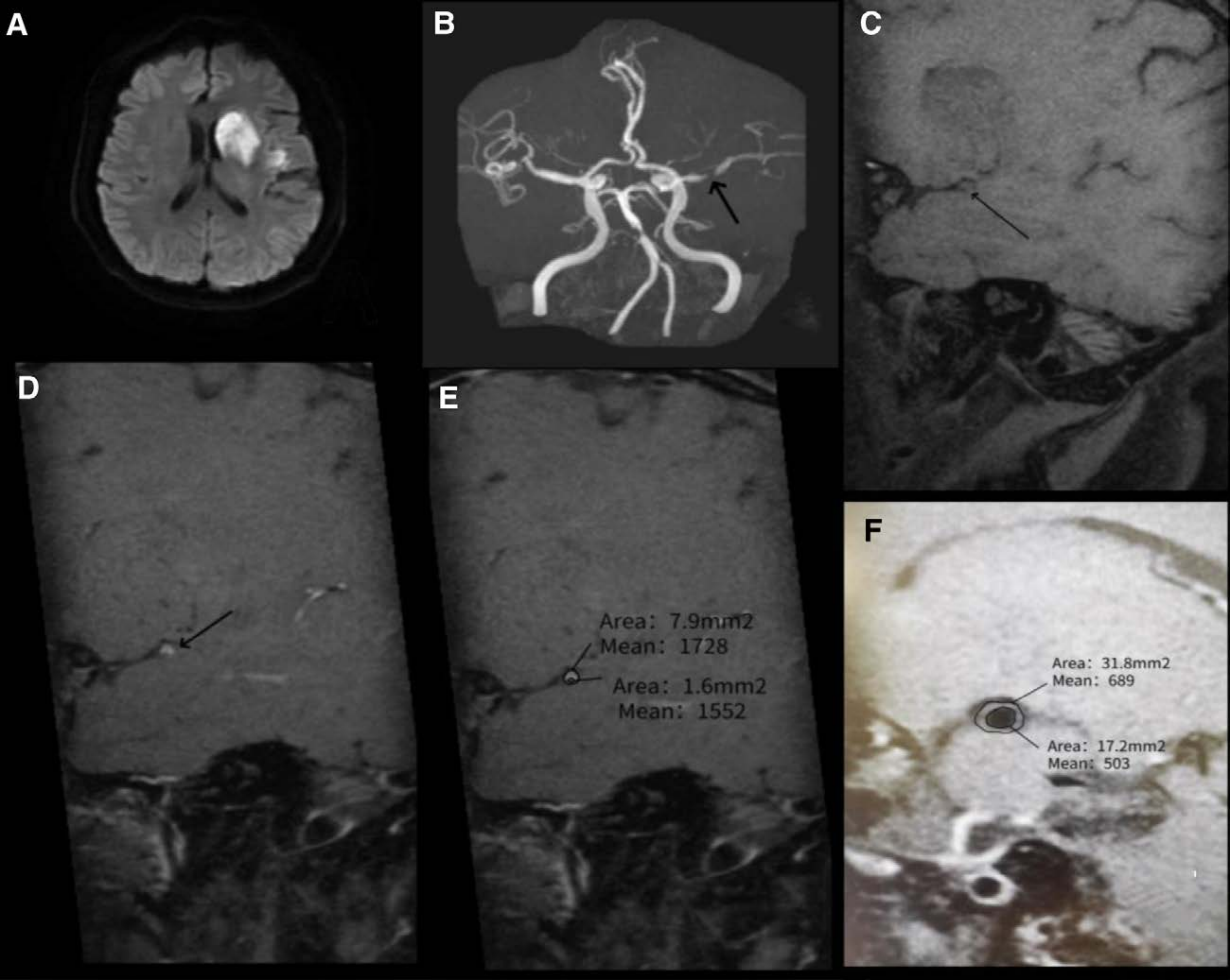
HR-MRI of a symptomatic MCA stenosis in a 54-yr-old male who presented with dysphonia for 1 d and right limb weakness for 10 h. (A) DWI indicates an AIS in the distribution of the left MCA. (B) TOF-MRA demonstrates severe stenosis of the M1 segment of the left MCA (white arrow). (C) The plaque is visible on T1-weighted HR-MRI (black arrow). (D) T1-weighted post-contrast HR-MRI shows eccentric wall thickening with enhancement at the plaque site (black arrow). (E) At the most narrowed site, post-contrast T1-weighted HR-MRI shows a vessel area of 7.9 mm^2^ and an LA of 1.6 mm^2^. (F) At the reference site, the vessel area is 31.8 mm^2^ and the LA is 17.2 mm^2^. The degree of stenosis = (1–1.6 mm^2^/ 17.2 mm^2^) × 100% = 90.7%. AIS = acute ischemic stroke, DWI = diffusion weighted imaging, HR-MRI = high-resolution magnetic resonance imaging, LA = lumen area, MCA = middle cerebral artery, TOF-MRA = time-of-flight magnetic resonance angiography.

**Figure 3.**
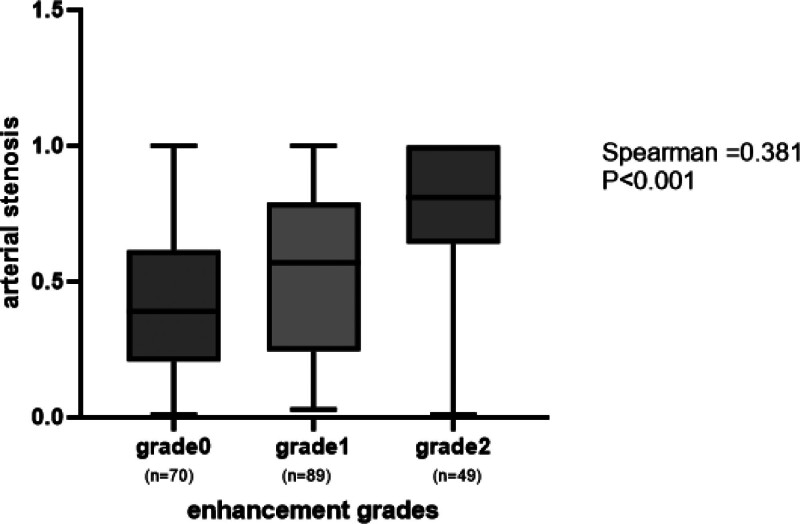
Tendency test of PE grade and AS severity in ICAS. AS = arterial stenosis, ICAS = intracranial atherosclerosis, PE = plaque enhancement.

Among the 208 patients, 70 individuals showed no arterial lesions with enhancement, while the remaining 138 patients exhibited varying levels of enhancement on HR-MRI. Of these, 89 patients had mild to moderate enhancement, and 49 patients displayed strong enhancement. Patients diagnosed with PE displayed a significantly greater plaque burden (*P* < .001) and more significant luminal stenosis (*P* < .001) in comparison to those without PE. Wall enhancement patterns observed in each group on HR-MRI are illustrated in Figures 1 and 2. At the most narrowed lumen, patients without PE were more likely to have larger VA and LA (*P* = .01, *P* < .001) (Table 1). Correlation analysis revealed a weak association between the severity of arterial stenosis (AS) and the grade of PE (Spearman *R* = 0.381, *P* < .001), suggesting that greater PE tends to accompany more severe stenosis (Fig. 3).

**Table 1:** Comparison of plaque characteristics in patients with and without enhancement.

	Wall enhancement (+) (n = 138)	Wall enhancement (−) (n = 70)	*P* value
VA reference (mm^2^)	17.1 (13.7–22)	19.2 (13.5–25.3)	.22
LA reference (mm^2^)	9.3 (7.3–12.2)	11 (7.7–13.5)	.22
VA MNL (mm^2^)	12.35 (8.2–19.7)	15.75 (10.9–23)	.01*
LA MNL (mm^2^)	2.7 (1.5–6.3)	6 (3–8.8)	<.001*
WA MNL(mm^2^)	8.5 (6.3–13.1)	9.85 (6.4–15.2)	.31
Plaque burden	0.76 (0.61–0.88)	0.62 (0.55–0.74)	<.001*
Luminal stenosis, (%)	0.71 (0.38–0.85)	0.39 (0.21–0.61)	<.001*

Data are represented as a number (%) or mean ± SD.

LA = lumen area, MNL = most narrowed lumen, Reference = reference site, SD = standard deviation, VA = vessel area, WA = wall area.

* *P* < .05.

The logistic regression analysis displayed that premorbid statin use was independently correlated with intracranial PE. (odds ratio: 2.14, 95% confidence interval: 1.01–4.56) (Table 2).

**Table 2:** Factors for enhancement of ICAS on HR-MRI.

	Total (n = 208)	Wall enhancement(+) (n = 138)	Wall enhancement (−) (n = 70)	Univariable analysis		Multivariable analysis	
				OR (95% CI)	*P* value	OR (95% CI)	*P* value
Mean age, year	57 ± 12.5	56.4 ± 13.06	58.1 ± 11.31	0.99 (0.97–1.01)	.36	–	–
Male sex	149 (71.6)	95 (68.8)	54 (77.1)	0.66 (0.34–1.27)	.21	–	–
BMI, kg/m^2^	25.67(23.71–27.68)	25.52 (23.66–27.68)	26.17 (24.22–28.91)	0.96 (0.86–1.04)	.34	–	–
Hypertension	132 (63.5)	89 (64.5)	43 (61.4)	0.89 (0.45–1.59)	.67	–	–
Diabetes	64 (30.8)	38 (27.5)	26 (37.1)	1.56 (0.84–2.87)	.16	–	–
Prior statin use	34 (16.3)	17 (12.3)	17 (24.3)	2.28 (1.08–4.81)	.03	2.14 (1.01–4.56)	.04
Current smoker, n (%)	77 (37)	54 (39.1)	23 (32.9)	0.76 (0.42–1.39)	.38	–	–
Albumin, g/L	39.3 (37.6–41.6)	39.4 (37.7–41.8)	38.9 (37.5–41)	1.04 (0.99–1.13)	.35	–	–
Protein level, g/L	65.6 (61.6–69.6)	65.9 (61.4–69.6)	64.9 (62–68.5)	1.02 (0.97–1.06)	.48	–	–
Total cholesterol, mmol/L	4.41 (3.6–5.18)	4.38 (3.57–5.2)	4.6 (3.64–5.1)	1.20 (0.94–1.53)	.14	–	–
Triglyceride, mmol/L	1.35 (3.6–5.18)	1.47 (1.03–1.98)	1.17 (0.99–1.9)	1.40 (0.97–2.03)	.03	1.36 (0.94–1.96)	.10
HDL cholesterol, mmol/L	1.02 (0.88–1.18)	1.02 (0.88–1.19)	1.02 (0.86–1.14)	1.69 (0.54–5.31)	.372	–	–
LDL cholesterol, mmol/L	2.9 (2.27–3.46)	2.91 (2.27–3.45)	2.82 (2.22–3.45)	1.36 (0.97–1.91)	.08	–	–
Non-HDL cholesterol, mmol/L	3.46 (2.74–4.08)	3.37 (2.74–4.11)	3.49 (2.72–3.99)	1.22 (0.92–1.62)	.16	–	–
Apo (a) lipoprotein, g/L	1.13 (0.96–1.3)	1.15 (1–1.34)	1.11 (0.95–1.26)	2.43 (0.72–8.25)	.15	–	–
Apo (b) lipoprotein, g/L	0.84 (0.69–0.99)	0.83 (0.71–1.03)	0.84 (0.65–0.98)	3.31 (0.95–11.524)	.06	–	–
Apo (b) lipoprotein/Apo (a) lipoprotein	0.74 (0.60–0.9)	0.74 (0.60–0.88)	0.72 (0.59–0.87)	1.47 (0.45–4.86)	.53	–	–
Blood urea nitrogen, mmol/L	4.6 (3.8–5.48)	4.6 (3.9–5.3)	4.9 (3.8–5.5)	0.98 (0.79–1.20)	.83	–	–
Uric acid, µmol/L	302.25 (245.18–380.28)	320.7 (245.4–396.2)	290.6 (236.2–344.9)	1 (1–1.01)	.06	–	–
Creatinine, µmol/L	68.3 (57.08–78.18)	68.3 (59.2–77.4)	71 (54.4–81.5)	1 (0.99–1.02)	.75	–	–
Creatine kinase, µkat/L	70.6 (54.08–98.15)	72.7 (54–104.4)	71.2 (54.3–89.4)	1 (1–1.01)	.24	–	–
Homocysteine, µmol/L	14.8 (11.7–19.65)	14.75 (11.8–19.6)	15.1 (11.8–19.4)	0.994 (0.978–1.011)	.48	–	–
Fasting glucose, mmol/L	5.16 (4.58–6.33)	5.135 (4.48–6.46)	5.37 (4.7–6.28)	0.99 (0.87–1.125)	.88	–	–
Fibrinogen level, g/L	2.76 (2.42–3.15)	2.755 (2.43–3.2)	2.75 (2.42–3.03)	1.12 (0.75–1.66)	.59	–	–
D-dimer, nmol/L	0.25 (0.17–0.40)	0.23 (0.17–0.4)	0.27 (0.19–0.4)	1.26 (0.52–3.10)	.61	–	–
White blood cell count, 10^9^/L	7.08 (5.94–8.33)	7.12 (5.96–8.15)	7.14 (6.43–8.46)	1.06 (0.92–1.23)	.43	–	–
Hemoglobin, g/L	143 (132.25–153)	144 (134–154)	141 (131–153)	1.01 (1–1.03)	.07	–	–
Platelet count, 10^9^/L	235.5 (204–275.5)	247 (205–286)	229 (204–267)	1 (0.99–1.01)	.27	–	–
HbA1c,%	6.1 (5.6–7.48)	6 (5.6–7.3)	6.7 (5.7–7.6)	0.96 (0.81–1.14)	.66	–	–

Data are expressed as mean ± SD or n (%).

BMI = body mass index, CI = confidence interval, HbA1c = hemoglobin A1c, HDL = high-density lipoprotein, HR-MRI = high-resolution magnetic resonance imaging, ICAS = intracranial atherosclerosis, LDL = low-density lipoprotein, OR = odds ratio, SD = standard deviation.

### 3.2. Patient characteristics and HR-MRI findings in relation to premorbid statin use

Among 208 patients, 34 (16.35%) had a history of statin usage before experiencing the stroke event. The morphological features, such as luminal stenosis, remodeling index, plaque burden, LA, and VA, did not exhibit significant differences according to premorbid statin use. However, among the parameters reflecting plaque activity, the proportion of patients with PE was found to be lower in those with premorbid statin use (nonusers, 70%; statin users, 50%; *P* = .03). In comparison to patients without using statin, those with premorbid statin use had lower total cholesterol (*P* < .001), LDL-C (*P* *<* .001), high-density lipoprotein cholesterol (*P* = .02), Non-HDL cholesterol (*P* *<* .001), Apo (b) lipoprotein (*P* *<* .001), and Apo (b) lipoprotein/Apo (a) lipoprotein (*P* *<* .001) (Table 3).

**Table 3:** Patient characteristics and plaque characteristics grouped by premorbid statin use.

Variable	Non-statins user (n = 174)	Statin user (n = 34)	*P*
Total cholesterol, mmol/L	4.65 (3.96–5.34)	3.29 (2.79–4.1)	<.001
Triglyceride, mmol/L	1.39 (1.03–1.91)	1.10 (0.84–1.92)	.07
HDL cholesterol, mmol/L	1.03 (0.89–1.19)	0.92 (0.81–1.09)	.02
LDL cholesterol, mmol/L	3.01 (2.53–3.5)	1.95 (1.68–2.56)	<.001
Non-HDL cholesterol, mmol/L	3.63 (2.91–4.23)	2.47 (1.85–2.86)	<.001
Apo (a) lipoprotein, g/L	1.15 (0.97–1.31)	1.09 (0.88–1.25)	.18
Apo (b) lipoprotein, g/L	0.88 (0.74–1.04)	0.62 (0.53–0.75)	<.001
Apo (b) lipoprotein/Apo (a) lipoprotein	0.77 (0.63–0.92)	0.60 (0.53–0.70)	<.001
VA reference (mm^2^)	17.95 (13.7–23.7)	17.75 (13.1–20.8)	.42
LA reference (mm^2^)	9.8 (7.4–13.1)	9.5 (7.7–13.1)	.68
VA MNL (mm^2^)	14 (9.3–21.6)	13.75 (8.3–22.6)	.95
LA MNL (mm^2^)	3.4 (1.6–7.3)	4.75 (2.1–8.5)	.25
WA MNL(mm^2^)	8.9 (6.4–13.9)	8.5 (5.2–14.7)	.47
Plaque burden	0.72 (0.58–0.86)	0.67 (0.55–0.78)	.10
Luminal stenosis, (%)	0.61 (0.27–0.82)	0.44 (0.21–0.78)	.15
Remodeling index	0.80 (0.58–1.06)	0.82 (0.65–1.10)	.50
PE	121 (70%)	17 (50%)	.03

HDL = high-density lipoprotein, LA = lumen area, LDL = low-density lipoprotein, MNL = most narrowed lumen, PE = plaque enhancement, Reference = reference site, SD = standard deviation, VA = vessel area, WA = wall area,.

Number (%) or mean ± SD are given for presenting the data.

## 4. Discussion

The primary finding of this study is that pre-stroke statin use was independently associated with a reduced prevalence of PE on HR-MRI, suggesting a stabilizing effect of statins on intracranial atherosclerotic plaques. While enhanced plaques were generally linked with greater stenosis severity and plaque burden, our results emphasize that statin therapy may attenuate such enhancement, indicating a potential role in modulating plaque vulnerability. This aligns with previous studies in coronary and carotid arteries, where statins have been shown to suppress inflammatory activity and promote plaque stabilization. Furthermore, a comparison of the “statin groups” revealed that “premorbid statin use” was correlated with a reduced occurrence of “PE,” a marker of plaque instability.

This study is consistent with previous research on the mechanism of statin stabilization of coronary artery plaques.^[6–9]^ Similar findings have also been reported in studies of coronary and extracranial carotid atherosclerosis. In these vascular territories, statin therapy has been associated with reduced PE on vessel wall imaging and a shift toward more stable plaque morphology. The mechanisms underlying this effect are likely multifactorial. Statins lower circulating LDL-C and non-HDL cholesterol, thereby decreasing lipid accumulation within the arterial wall. In addition, their pleiotropic actions – such as reducing vascular inflammation, improving endothelial function, and inhibiting intraplaque neovascularization – may collectively contribute to attenuation of PE.^[9]^ These mechanisms provide a plausible explanation for our observation that patients with prior statin use exhibited a lower prevalence of PE in ICAS.

Accumulating evidence suggests that vessel wall enhancement on HR-MRI may serve as an indicator of vulnerable or symptomatic plaque, given its frequent association with multiple embolic infarctions. Results indicate that patients with PE are more likely to have “severe stenotic lesions.”^[10]^ It has also been reported that a higher proportion of patients with intracranial PE show positive findings on conventional MRA compared to those without PE.^[11,12]^ Furthermore, a positive correlation has been observed between the extent of PE and the severity of AS, suggesting that this could contribute to ischemic stroke due to enhanced plaques. The relationship between wall enhancement and the degree of AS exhibited a similar trend in our study.

Statins are known to provide a range of beneficial effects beyond lipid-lowering, including reducing systemic inflammation and improving endothelial dysfunction.^[13,14]^ In both the coronary and extracranial carotid arteries, statins have been shown to stabilize culprit atherosclerotic plaques.^[15,16]^ Recent investigations have shown that premorbid statin usage is connected to a lower frequency and volume of PE, hence lowering the risk of massive cerebral infarcts.^[17]^ Previous studies have shown that improving HR-MRI-detected lesions could indicate underlying plaque bleeding, neovascularisation, or inflammatory activity.^[18]^ Furthermore, lipid metabolism has been recognized as a key contributor to plaque formation.^[19]^ Several studies have shown that lipid-related markers like non-HDL cholesterol, total cholesterol, LDL-C, and the ApoB/ApoA lipoprotein ratio influence ICAS load.^[20–22]^ Moreover, independent and important predictors of PE on HR-MRI have included LDL-C, triglycerides, non-HDL cholesterol, and ApoB. These results suggest that ICAS improvement is influenced by lipid metabolism and that strong lipid-lowering policies, including rigorous statin treatment, could lessen the risk of forming susceptible plaques.

Our results provide further evidence for the hypothesis that previous statin usage is independently related to PE in ICAS. Patients who had used statins before to stroke started showed also less PE incidence.

Its retrospective cross-sectional methodology and small sample size drawn from a single stroke center are among the study’s limitations, which may restrict the generalizability of the findings. Second, we only focused on the characteristics of culprit plaques and did not assess asymptomatic plaques. Third, quantitative measurement of the enhanced signal intensity of arterial plaques was not performed. Fourth, because only a small proportion of patients had received statin therapy before stroke, we were unable to conduct dose- or class-specific analyses. In addition, potential confounding factors related to statin use, such as medication compliance, treatment duration, and baseline vascular risk differences between statin users and non-users, could not be fully controlled in this study. These factors may have influenced the observed association between statin use and PE. Our goal is to address these shortcomings in future prospective, multicenter studies with larger sample sizes.

In conclusion, a strong association was noted between premorbid statin intake and a lower incidence of PE. Moreover, there was a significant relationship between the magnitude of enhancement in ICAS and the severity of stenosis. This study highlights HR-MRI’s usefulness in characterizing plaques and providing accurate direction for clinical decision-making.

## Author contributions

**Conceptualization:** Shibo Dong, Hongshan Chu, Yuan Zhang, Ruisheng Duan, Hongyu Hao, Xing Xing, Ya Gao.

**Data curation:** Shibo Dong, Ruisheng Duan, Jin An.

**Formal analysis:** Shibo Dong, Hongshan Chu, Xing Xing, Jin An.

**Investigation:** Shibo Dong, Hongshan Chu, Yuan Zhang, Ruisheng Duan, Hongyu Hao, Xing Xing, Ya Gao, Xiangjian Xiao.

**Methodology:** Yuan Zhang, Ruisheng Duan, Hongyu Hao, Xiangjian Xiao.

**Resources:** Xiangjian Xiao.

**Software:** Nan Yin.

**Supervision:** Hongshan Chu, Xing Xing, Nan Yin, Jin An, Ya Gao.

**Validation:** Shibo Dong, Hongshan Chu, Yuan Zhang, Hongyu Hao, Xing Xing, Nan Yin.

**Visualization:** Shibo Dong, Hongshan Chu, Hongyu Hao, Xiangjian Xiao.

**Writing – original draft:** Shibo Dong, Hongshan Chu.

**Writing – review & editing:** Shibo Dong.
